# Carlos Gutiérrez-Merino: Synergy of Theory and Experimentation in Biological Membrane Research

**DOI:** 10.3390/molecules29040820

**Published:** 2024-02-10

**Authors:** Silvia S. Antollini, Francisco J. Barrantes

**Affiliations:** 1 Departamento de Biología, Bioquímica y Farmacia, Universidad Nacional del Sur, Instituto de Investigaciones Bioquímicas de Bahía Blanca (CONICET-UNS), Bahía Blanca 8000, Argentina; silviant@criba.edu.ar; 2 Laboratory of Molecular Neurobiology, BIOMED UCA-CONICET, Buenos Aires C1107AAZ, Argentina

**Keywords:** Förster resonance energy transfer (FRET), membrane biophysics, membrane proteins, membrane lipids, lipid–protein interactions, nicotinic acetylcholine receptor

## Abstract

Professor Carlos Gutiérrez-Merino, a prominent scientist working in the complex realm of biological membranes, has made significant theoretical and experimental contributions to the field. Contemporaneous with the development of the fluid-mosaic model of Singer and Nicolson, the Förster resonance energy transfer (FRET) approach has become an invaluable tool for studying molecular interactions in membranes, providing structural insights on a scale of 1–10 nm and remaining important alongside evolving perspectives on membrane structures. In the last few decades, Gutiérrez-Merino’s work has covered multiple facets in the field of FRET, with his contributions producing significant advances in quantitative membrane biology. His more recent experimental work expanded the ground concepts of FRET to high-resolution cell imaging. Commencing in the late 1980s, a series of collaborations between Gutiérrez-Merino and the authors involved research visits and joint investigations focused on the nicotinic acetylcholine receptor and its relation to membrane lipids, fostering a lasting friendship.

## 1. Introduction

Professor Carlos Gutiérrez-Merino belongs to a group of curious and restless scientists who have contributed with incisive theoretical approaches and solid experimental work to the advancement of our knowledge in the highly complex and competitive field of biological membranes. He first approached us in the late 1980s, leading to a fruitful scientific collaboration entailing several research visits to carry out experimental work at the Institute for Biochemical Research of the Universidad Nacional del Sur in Bahía Blanca, Argentina, and reciprocated by visits of F.J.B. to Prof. Gutiérrez-Merino’s laboratory at the Department of Biochemistry and Molecular Biology of the University of Extremadura in Spain, catalyzed by the Cooperation Programme with Iberoamerica. These exchanges extended throughout two decades, resulted in the publication of eight research papers, and helped forge a strong friendship with Carlos, a cultivated and warm-hearted individual who generously devoted many hours to teaching and holding discussions with research students and members of staff in our laboratory.

Biological membranes are complex and dynamic and remain the subject of multiple controversies. Various theories and models were suggested before Singer and Nicolson proposed the fluid-mosaic model in 1972 [1]. The innovative depiction of a biological membrane of the Singer and Nicolson model gained wide acceptance, provoking ample discussions, triggering experiments, and ultimately shedding light on membrane structure and function. The model has withstood the test of time, with various modifications and extensions, despite the enormous amount of new information gained during the last decades, some of which openly challenged the fluid-mosaic depiction [2,3,4,5]. 

Currently, a biological membrane is conceptualized as an intricate, crowded structure with a diverse lipid and protein composition, featuring lateral and transverse asymmetry, variable patchiness, variable thickness, and high protein occupancy [2,5]. It is universally accepted that biological membranes act as barriers that separate two fluid media, preventing direct contact between the inner and outer compartments. However, constituting a physical barrier is not their sole function. Many essential biochemical reactions for cell life, involving metabolic and signaling processes with membrane-bound enzymes and transmembrane proteins such as G-protein coupled receptors (e.g., rhodopsin or muscarinic receptors) and ion channels (e.g., nicotinic, histaminergic, GABAergic, or glutamatergic receptors), take place in cell membranes. This makes membranes pivotal scenarios in nearly all cellular physiological and pathological processes. These essential reactions require molecular communication, involving both protein–protein and protein–lipid interactions, and the study of these processes posed formidable experimental challenges a few decades ago. 

## 2. Gutiérrez-Merino’s Development of Theoretical Approaches in Fluorescence Spectroscopy in Biological Membrane Research

At the time when the fluid-mosaic membrane model was being developed, Förster resonance energy transfer (FRET) emerged as a revolutionary and extremely useful technique for the study of molecular interactions in biological systems, as it allows the obtention of structural details in the 1–10 nm scale size [6,7,8]. FRET theoretical developments addressing the analysis of experimental data permitted biophysicists to extract quantitative information about a great variety of membrane properties [9,10,11,12,13,14,15,16,17,18,19,20,21,22,23,24,25,26].

One of Gutiérrez-Merino´s contributions to the field of FRET took the form of two theoretical approaches that were presented almost simultaneously. One of these approaches was developed for model systems of binary lipid mixtures undergoing phase separation and involving four main conditions: (a) a triangular network of lipids in a gel phase [27]; (b) an insignificant relative population of small clusters; (c) donor and acceptor molecules oriented in the plane of the membrane (achieved at very low concentrations of labeled lipids, typically <5%); and (d) a substantial number of acceptor molecules for each donor molecule [17]. The acceptor molecules were assumed to be situated in concentric layers of lipid (discs) around a single donor molecule.

The rate of energy transfer (k_r_) between a donor and an acceptor separated by a distance r was calculated using the value of K_T_(r) obtained with Förster´s equation [28], and the relative number of phospholipid molecules in each disc with respect to the number of phospholipid molecules in the first disc around a given molecule (Equation (1)) was formulated as follows:

k_T_ (r) = τ_o_^−1^ (R_0_/r)^−6^    with k_r_ = [k_T_ (r)/k_T_ (r_1_)] n/6 (1)

where τ_o_ is the lifetime of the donor in the absence of the acceptor; R_0_ is the distance in Å between donor and acceptor molecules at which the transfer efficiency is 50%; r_1_ is the average intermolecular distance of the triangular lattice; and n is the number of phospholipid molecules in each disc surrounding a given phospholipid molecule. Considering the random distribution of donor and acceptor molecules in the plane of the membrane, it is assumed that no change takes place in the orientation factor κ^2^ (a parameter used in the calculation of R_0_) in the passage from one lipid disc to the next [17].

Considering the rate of energy transfer, theoretical analyses were developed for a binary, partially mixed lipid bilayer undergoing lateral phase separation (Equation (6)), intended to represent a realistic condition met in biomembranes [17]. The root of this reasoning stems from the analysis of both an ideal mixture of lipids (Equation (2)) and a completely immiscible mixture of lipids (Equations (3) and (4)). In both cases, the experimental data were obtained from unilamellar vesicles, whereby the bilayer curvature was considered negligible.

k_T_ = [1 − N_A_/(N_A_ + N_B_)] f_a_ k_T_(r_1_) (2)

where N_A_ and N_B_ are the number of molecules of randomly distributed donor and acceptor lipids, respectively, and f_a_ is the fraction of B molecules.

k_T_ = [(2n + 6)/6n] f_a_ k_T_(r_1_) (3)

where n is the number of lipid molecules in a cluster consisting of up to six molecules of the minority component in the lipid mixture, with completely immiscible A and B molecules, and

k_T_ = (6n)^−1^ {6 + 12 [s + n_exc_/6 (s + 1)]} f_a_ k_T_(r_1_) (4)

where s is the number of complete lipid shells of the cluster, which is an integer ≥ 1, and n_exc_ is the number of lipid molecules in the incomplete outer shell of the cluster.

Next, the rate of energy transfer for a binary partially mixed lipid bilayer undergoing lateral phase separation is calculated, as follows:

k_T_ = f_a_ k_T_(r_1_) 〈i〉^−1^ (〈1/i〉 + 2〈s_i_/i〉) (5)

where i corresponds to lipid molecules of class A, and 〈i〉 and 〈s_i_/i〉 are the average cluster size in terms of number of lipid molecules within and the ratio of cluster shells to lipid molecules in the cluster, respectively. Equation (5) can be reformulated by expressing the last term as 〈(1 + 2s_i_)/i〉 = ½ 〈D_i_/i〉, where D_i_ is the diameter of the circular cluster i, and 〈D_i_/i〉 = 〈D_i_−1〉. Thus,

k_T_ = ½ f_a_ k_T_(r_1_) 〈i〉^−1^ 〈D_i_^−1^〉 (6)

This allows one to calculate the average cluster size of the minority lipid (lipid A).

In summary, the average energy transfer efficiency provides a means to ascertain, directly from experimental observations, the average size of lipid clusters, correlating this information with the concentrations of both lipids in the mixture, within constrained molar fraction ranges. These novel contributions from Gutiérrez-Merino significantly contributed to enhancing our understanding of the thermodynamic principles governing lateral phase separation in lipid membranes [17].

The scope of this analysis was broadened to encompass additional conditions, in particular the inclusion of membrane proteins, as addressed in the second theoretical approach based on FRET, which Gutiérrez-Merino introduced more than forty years ago [18]. In this scenario, the assumption (a) of the first approach required modifications to incorporate the distribution and state of aggregation of integral membrane proteins. Again, FRET experiments conducted on these more complex biological systems were key to reaching the conclusion that the average rate of energy transfer could function as a quantitative ruler of the following, among other metrics of membrane properties: (a) transmembrane distances (i) between proteins or (ii) between proteins and lipids; (b) distances between sites within a single protein; (c) changes in protein aggregation; (d) lipid heterogeneities; and (e) random or non-random protein distribution. Measurements of the average rate of energy transfer between protein and phospholipid molecules, labeled with donor and acceptor molecules, respectively, were often used in these experiments. The ability to accurately characterize the above properties and processes strongly depends on the careful selection of donor and acceptor molecules. For example, in the examination of random and non-random protein distributions in a membrane, it is imperative to employ fluorescently labeled proteins for the donor–acceptor pair. To investigate protein lateral phase separation from lipids, it is usually necessary to resort to a donor-labeled protein and acceptor-labeled lipids. In the latter case, it is crucial to verify that the fluorescent label of the lipids does not exclude them from the immediate perimeter of the protein or induce non-preferential binding to the protein of interest [18].

**Figure 1:**
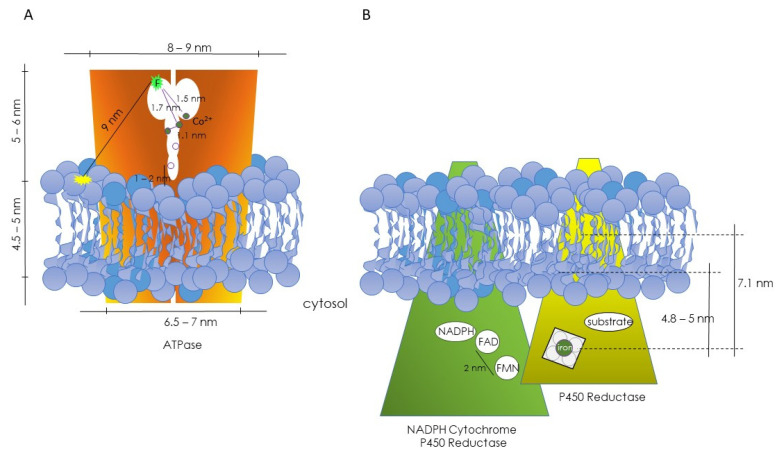
Diagrams depicting the hypothetical topographical relationships between the ATPase (**A**) and NADPH cytochrome P450 reductase and P450 reductase (**B**) with their respective lipid microenvironments. In both cases, information was obtained through FRET studies. (**A**) From Gutiérrez-Merino et al., refs. [29,30]. (**B**) From Centeno and Gutiérrez-Merino, ref. [31].

The above theoretical approach was applied to the study of the (Ca^2+^-Mg^2+^) ATPase. In this case, ATPase labeled with a fluorescein tag at the ATP binding site served as the donor, and rhodamine-labeled lipids acted as acceptors. This configuration allowed the fluorescently labeled ATP binding site of the enzyme to sit at a distance from the lipid–water interface of the membrane. Moreover, this series of experiments made it possible to postulate that the (Ca^2+^-Mg^2+^) ATPase existed as a dimer, with the ATP binding sites of each monomer located close to the protein–protein interface [29]. Subsequently, using Co^2+^ as an acceptor for the fluorescence emitted by fluorescein and employing the same theoretical approach, it was possible to establish the location of functional binding sites, in particular the regulatory metal ion sites for free Co^2+^, which likely correlate with Ca^2+^ sites (Figure 1A) [30].

In 1994, Gutiérrez-Merino and colleagues [32] extensively discussed the utilization of FRET for measuring distances between donor and acceptor molecules, building upon their prior expertise [29,30,31,33]. As is now well established, this nonradiative process involves the transfer of excitation energy through long-range dipole –dipole coupling, a phenomenon affected by the distance between the two molecules and their orientation [28]. A significant cautionary note was issued regarding the precision of such measurements. Obtaining a single donor–acceptor pair in membrane systems is challenging unless very restrictive conditions are met, such as an R_0_ value smaller than half the dimension of the protein diameter or a homogeneous dispersion of monomeric membrane proteins with substantial “dilution” in a lipid matrix. Hence, calculations of distances between two functional protein sites or a protein site and a lipid pose considerable uncertainty.

A similar reasoning was employed to investigate the positioning of functional centers in the microsomal cytochrome P450 system [31]. Energy-transfer studies enabled the determination of the locations of the two prosthetic groups (FAD and FMN) of NADPH-cytochrome P450 reductase, as well as the heme group and the substrate binding site of cytochrome P450 (Figure 1B). The estimated distance between the FAD and FMN groups (donor–acceptor pair) was approximately 2 nm. Using a lipid labeled with rhodamine (RPE) as an acceptor for the reductase’s fluorescence, the position of the flavins was estimated to be more than 5 nm from the rhodamine group. Consequently, it was concluded that the FMN and FAD groups are not exposed to the enzyme’s surface and are distant from the lipid–water interface, with FAD more deeply buried than FMN in the enzyme’s 3D structure. In the case of cytochrome P450, two different donor–acceptor pairs were used: diphenylhexatriene (DPH)–heme and 4H7HC–heme, with DPH located deep inside the lipid bilayer, and the coumarin group of 4H7HC close to the polar headgroup. Distances of 7.1 nm between the heme and DPH groups and of approximately 4.8 nm from the heme group to the lipid–water interface were obtained. A third donor–acceptor pair was used (ethoxycoumarin–heme group). Ethoxycoumarin is a good substrate for these isoenzymes, and a distance of 5.3 nm between the heme group and 7-ethoxycoumarin could be calculated (Figure 1B) [31].

## 3. Gutiérrez-Merino’s Incursions into the Field of Nicotinic Acetylcholine Receptor–Lipid Interactions

The influence of lipids on the conformation, function, and topography in the membrane of the nicotinic acetylcholine receptor (nAChR) has been the subject of intense study for the last 50 years [34]. Synthesis, assembly, and function of the nAChR strongly depend on the properties and characteristics of the membrane in which it is embedded. The layer of lipids in the immediate vicinity of the receptor has distinct properties relative to bulk lipids. Different experimental strategies have been used over the last decades to investigate receptor–lipid interactions. Fluorescence spectroscopy and fluorescence microscopy have occupied a central position in characterizing many of the properties of the nAChR that we now know of. Carlos Gutiérrez-Merino contributed to this endeavor during his collaboration with our laboratory in the early 1990s. In this Section, we will summarize some of these studies.

nAChRs are pentameric integral membrane proteins belonging to the Cys-loop superfamily of ligand-gated ion channels [35,36,37,38]. Each of its five subunits possesses a large extracellular region that harbors the agonist-binding site, a transmembrane region with extensive contacts with surrounding lipids through evolutionarily conserved structural motifs [34,39,40,41] and an intracellular region containing modulatory sites and determinants of channel conductance [42,43]. The transmembrane domain consists of four segments (TM1–TM4), with TM2 forming the walls of the ion channel pore and TM1, TM3, and TM4 being more externally located [34,44,45]. Among these, TM4, the most peripheral transmembrane domain, has the closest contact with membrane lipids and constitutes the lipid-sensing domain of the protein [46,47].

The nAChR is a typical transmembrane protein, and the properties and characteristics of the membrane in which it resides are therefore important influences on its function, biosynthesis, and proper assembly [36,46,47,48,49,50]. Reciprocally, the nAChR exerts influence on its neighboring lipids [51,52,53]. The muscle-type nAChR at the neuromuscular junction and the electric fish of electromotor synapses (electric eels and electric rays) is enveloped by a layer of interstitial lipids, relatively immobilized in the microsecond time-window of electron spin resonance (ESR) spectroscopy, with distinct features relative to bulk lipids [51]. Thus, the lipid molecules closely associated with the protein exhibit a slow exchange rate with bulk lipids. Mobile lipids interact with membrane proteins in a relatively less specific manner and exhibit a faster rate of exchange with bulk lipids [54,55,56,57]. It has been stressed that in contrast to other biological membranes, the postsynaptic membrane in which the nAChR is embedded is a unique system, whereby the amount of bulk lipid in the tight 2-dimensional lattice of receptor proteins is minimal, with only a few layers of interstitial lipid in between adjacent receptor molecules, and consequently with little, if any, “bulk” lipid [34].

Since lipid composition undergoes changes with aging and in response to various neurodegenerative diseases [58,59], and bearing in mind that lipid content varies across different tissues, it becomes imperative to comprehend how alterations in the nAChR lipid environment impact its structure, activity, and dysfunction.

One key aspect of biological membranes is their heterogeneity, which comprises both lateral and transbilayer asymmetries, two topographical properties that have an impact on integral membrane proteins. The aminophospholipids phosphatidylethanolamine (PE) and phosphatidylserine (PS), along with phosphatidylinositol, are primarily situated in the inner leaflet of the plasmalemma, while phosphatidylcholine (PC) and sphingomyelin (SM) are predominantly located in the outer leaflet in most mammalian cells [60,61]. In mouse synaptic plasma membranes, the outer monolayer exhibits greater fluidity than the inner leaflet [62,63], a phenomenon that correlates with the notable prevalence (88%) of cholesterol in the inner, cytoplasmic hemilayer in some biological membranes [64]. In one of Gutiérrez-Merino’s visits to our laboratory, we explored lipid asymmetry in native nAChR-rich membranes from *Torpedo* electrocytes. The dimensions of a lipid bilayer, typically ranging from 4 to 5 nm [65,66], fall within the same range as the R_0_ observed between different pairs of donor and acceptor fluorescent molecules commonly employed in biophysical studies of biological membranes. Hence, it was predicted that the efficiency of FRET between lipids labeled as donor and acceptor in opposite leaflets of the membrane bilayer would be significantly lower than the transfer between donor and acceptor molecules located on the same membrane hemilayer. We used lipids tagged with an NBD group as donor, labeled at various positions such as the headgroup, C6, and C12. The fluorescent probe rhodamine-PE (N-Rho-PE) was chosen as the acceptor. A calculated R_0_ value of 5.58 nm for the donor–acceptor pair was determined [67]. We concluded that the phospholipids PC and PE were primarily located in the exofacial leaflet in nAChR-enriched membranes from *Torpedo*. Additionally, the evaluation of energy transfer efficiency reported deviations from a uniform distribution of the labelled lipids within this leaflet when high molar ratios of acceptor lipids were used in liposomes prepared with the endogenous PC fraction extracted from native nAChR-rich membrane samples. 

In a subsequent study, we addressed the precise location of SM in native nAChR-rich membranes from *Torpedo marmorata*. At that time, the distribution of SM on the cell-surface membrane was not known, nor was it clear whether this lipid held any structural and/or functional significance relative to the major functional molecule in this membrane, the nAChR. Previous work had shown the enrichment of SM in membrane regions where nAChR clusters were present. The lipid composition of these regions differed from the bulk membrane composition [68]. In nAChR-rich membranes derived from the electric organ of Torpedinidae species, the primary phospholipids were PC (40%), PE (35%), and PS (13%), with a much lower SM content (~5% of the total phospholipid content) [69,70]. Using classical biochemical approaches, it was possible to conclude that SM in native nAChR-rich membranes from *T. marmorata* was enriched in the outer membrane hemilayer, confirming the transbilayer asymmetry of this lipid. However, this did not imply homogeneity in the distribution of SM in the external hemilayer. To address this question, N-[10-(1-pyrenyl)decanoyl] sphingomyelin (Py-SM) was used as a reporter group of the lipid physical state. FRET was also employed to assess the spatial relationship between the receptor protein and the fluorescent lipid analogue in the immediate vicinity of the nAChR protein as well as to determine its affinity for the receptor.

The ratio of excited-state pyrene dimer (excimer) to monomer Py-SM fluorescence (FE–FM) provides insight into the intermolecular collisional frequency of these fluorophores; hence, it constitutes a parameter directly affected by probe concentration. The increased rate in the FE–FM ratio as a function of Py-SM concentration was twofold higher under FRET conditions than under direct excitation of the probe. This observation suggested a preferential partitioning of the Py-SM analogue in the protein-adjacent region. Applying one of Gutiérrez-Merino´s theoretical FRET approaches [17,18] to the experimental data enabled us to determine R_0_. A value of 21 ± 2 Å was obtained, in good agreement with previous reports [71]. The new data permitted the calculation of the k_r_ for SM in the nAChR vicinity; a value of 0.55 relative to the average bulk lipid moiety in the membrane was obtained. This result suggested that Py-SM displayed a moderate affinity for the membrane-bound nAChR. Treatment of the membrane with sphingomyelinase converts SM into phosphorylcholine and ceramide (Cer). In intact membranes, enzymatic hydrolysis removes only outer leaflet SMs [72]. When nAChR-rich membranes were treated with sphingomyelinase and FRET efficiency was measured before and after enzymatic hydrolysis, a noticeable increase in FRET efficiency between the protein and the resulting Py-Cer was observed, indicating an enhanced affinity of Py-Cer for the donor protein and/or greater accessibility of this lipid to the lipid microenvironment of the nAChR relative to the Py-SM precursor. Additionally, a gradual decrease in the FE–FM ratio was observed during enzymatic digestion, reinforcing the idea that Py-Cer exhibits higher affinity and/or greater accessibility to the lipid–protein interface than Py-SM. This finding could be rationalized in terms of the structural similarity between Cer and free fatty acids (FFAs), since the latter exhibit approximately four times greater affinity for the membrane-bound *Torpedo* nAChR relative to PC [73,74]. Considering a total of 44 lipids comprising the nAChR vicinal lipid, only 2.2 molecules of SM were estimated to be at the nAChR–lipid interface [75]. If one considers that the conversion of SM to Cer, a process that occurs naturally and has significant functional consequences, could produce an increase in Cer molecules at the nAChR–lipid interface, it also implies a relative decrease in other lipids in the receptor microenvironment. These changes in the lipid–nAChR interface might entail functional consequences, given the well-known influence of lipids on the conformation and allosteric mechanisms of this receptor [34].

To learn about the physical characteristics of the membrane in which the nAChR is inserted and of the lipid belt region, we resorted to the amphiphilic fluorescent probe Laurdan (6-dodecanoyl-2-dimethylaminonaphthalene) [76]. Laurdan, one of the several solvatochromic probes conceived and synthesized by Gregorio Weber and colleagues, possesses an exquisite spectral sensitivity to the phase state of the membrane because of its capacity to sense the polarity and the molecular dynamics of dipoles in its surroundings, due to the effect of dipolar relaxation processes [77,78,79]. Laurdan localizes at the hydrophilic–hydrophobic interface of the lipid bilayer [80,81], with its lauric acid moiety at the phospholipid acyl chain region and its naphthalene moiety at the level of the phospholipid glycerol backbone. The so-called general polarization (GP) of Laurdan, a ratiometric fluorescence technique, exploits its advantageous spectral properties, as it was initially developed for time-resolved fluorescence emission spectral analysis in cuvette studies [76].

**Figure 2:**
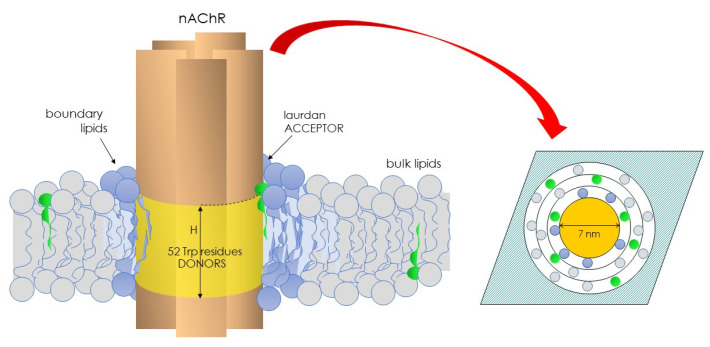
Illustration depicting the nAChR and its 52 Trp residues and the vicinal, boundary lipid of the Laurdan molecules’ (green) partition. nAChR Trp residues act as donors, and the solvatochromic Laurdan molecules as acceptors. This pair constitutes the basis of the FRET-GP concept introduced in the field of biological membranes in 1996 (ref. [52]).

To learn about the physical state of the lipid microenvironment of the nAChR, we conceived a novel approach in Laurdan studies that relied on exploiting the Förster energy transfer from the intrinsic fluorescence of the nAChR (donor) to Laurdan as the acceptor, thus introducing FRET-GP in membrane biophysics [52]. As shown in Figure 2, the nAChR has 52 tryptophan residues, 51 at the transmembrane region and 1 at the extracellular domain. Using the structural data available at that time [82], we reasoned that the transmembrane Trp residues were arranged as an interconnected network in a ring-like three-dimensional structure with an outer radius of 32.5 Å. Furthermore, in view of the long-axis dimensions of the nAChR molecule and the width of the lipid bilayer [82], the height of the plane of nAChR tryptophan residues was allowed to vary between 0 and 50 Å (Figure 2). The nAChR cylinder was in turn assumed to be surrounded by a belt of lipid molecules of approximately 10 Å diameter each. The plane of acceptors was calculated assuming an area per lipid molecule of 0.75 nm^2^ [83].

**Figure 3:**
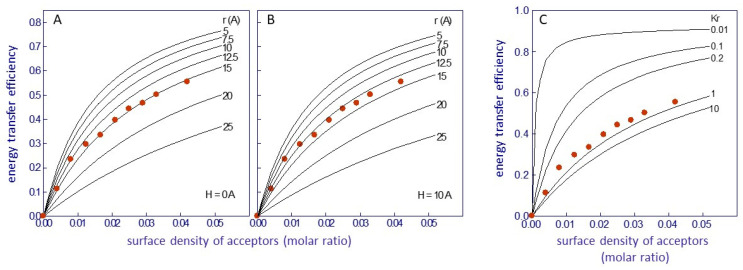
Efficacy of Förster resonance energy transfer (FRET) between the protein intrinsic fluorescence (nAChR) and Laurdan as a function of the surface density of energy transfer acceptors, in nAChR-rich membranes from *T. marmorata*. The continuous lines correspond to theoretical curves representing (**A**,**B**) minimal distances (r) between donor and acceptor, calculated utilizing the methodology proposed by Gutierrez-Merino [18], with R_0_ = 29 Å and H set at 0 Å and 10 Å, respectively, and (**C**) different values of the apparent dissociation constant of Laurdan for the boundary lipids (Kr), calculated using the treatment of Gutiérrez-Merino et al. [29], with H = 0 Å, r = 15 Å, and R_0_ = 29 Å. Data taken from ref. [52].

The parameter H was used as a measure of the distance between donor–acceptor planes normal to the membrane surface. Several conditions were considered [29]: (i) neighboring nAChR molecules did not generate an accessible surface area to acceptor molecules; (ii) the homotransfer of energy was allowed to occur between Trp residues; and (iii) a distance of closest approach (r) of 10 Å corresponding to the sum of the van der Waal radii of Trp and Laurdan was considered. The distribution (random or nonrandom) of Laurdan in the nAChR-vicinal lipid was calculated using the parameter α (Equation (7)), which considers the probability of occupancy of sites at the lipid belt region by Laurdan (L1) relative to unlabeled lipids (L2), as follows:

α = (x_1_/K_1_)/(x_2_/K_2_) = (x_1_/x_2_) K_r_^−1^ (7)

where x_1_ + x_2_ = 1, and K_r_ is the apparent dissociation constant of Laurdan for the lipid belt region. A value of K_r_ = 1 implies random distribution of the probe in the membrane, whereas values less than or greater than 1 imply Laurdan´s preferential partition at the lipid belt region or Laurdan´s exclusion from this region, respectively. Theoretical fittings with varying R_0_, r, and H were performed and compared with the experimental data using a set of curves with different K_r_ values (from 0.01 to 100) with a fixed R_0_ (Figure 3) [52]. An R_0_ value of 29 ± 1 Å for an intrinsic fluorescence protein–Laurdan donor–acceptor pair was calculated. With this value, an average value of r = 14 + 1 Å was chosen for H between 0 and 10 Å (Figure 3A,B). This value corresponds to the thickness of a single layer of phospholipid molecules (0.75 nm^2^ in surface area; [83]), in agreement with the proposed location of Laurdan in the bilayer [78,80,81]. Finally, a value of K_r_ near 1 was obtained (Figure 3C), indicating a random distribution of Laurdan in the lipid bilayer, confirming previous observations that suggested that the location of Laurdan in the membrane was independent of the nature of the phospholipid polar headgroup [78].

In practice, theoretical and experimental FRET efficiency (E) are related as follows:

E = k_T_/k_0_ + k_T_ = 1 − ϕ/ϕ_D_ ≈ 1 − I/I_D_ (8)

where k_0_ is the rate of energy transfer, and donor and acceptor molecules are separated by R_0_; ϕ and ϕ_D_ are fluorescence quantum yields of the donor in the presence and absence of acceptor molecules, respectively; and I and I_D_ are the fluorescence emission intensity of the donor in the presence and absence of acceptor molecules, respectively.

The knowledge gained through these studies on the precise localization of Laurdan at the first shell of lipids surrounding the nAChR and the observation that the convenient positioning of Laurdan made it possible to use this probe as an acceptor of the nAChR intrinsic fluorescence in FRET-GP prompted us to undertake a series of studies on nAChR–lipid interactions in native membranes. Using fluorescence spectroscopy, we could identify sites for free fatty acids, various sterols, and phospholipids in the nAChR [84]. These sites were found to be preserved after controlled proteolysis of the extracellular nAChR moiety and were masked during nAChR desensitization [53]. Additionally, we utilized this fluorescent donor–acceptor pair to study nAChR localization in liquid-ordered and/or liquid-disordered domains in membranes of various compositions and asymmetries [85,86].

## 4. Gutiérrez-Merino’s Use of FRET in Imaging Studies of Biological Membranes

One of the most interesting aspects of FRET studies in membranes is the spatial selectivity, permitting the observation of phenomena restricted only to acceptor molecules that are just a few nanometers away from the donor molecule, thus providing high spatial resolution for the case of multiple acceptors per donor, a property that has been exploited in cell imaging.

Neuronal apoptosis is intimately related to oxidative stress [87], and a considerable amount of research has been undertaken in this field to understand not only physiological apoptosis but also that associated with pathophysiological conditions such as neurodegenerative diseases (i.e., Alzheimer disease [88]). Gutiérrez-Merino expanded the potential of FRET approaches by transitioning from cuvettes to the microscope, focusing on this challenging problem. Changes in the red/orange autofluorescence of flavoprotein cytochrome b5 reductase (Cb5R), a major component of the plasma membrane redox chain, and other flavoprotein oxidases also bound to membranes were observed under cellular oxidative stress conditions [89]. Thus, flavins were used as donor molecules to study their location in the plasma membrane. Two suitable acceptor molecules were *N*-(3-triethylammoniumpropyl)-4-(4-(4-(diethylamino)phenyl)butadienyl)-pyridinium dibromide (RH-414) and *N*-(3-triethylammoniumpropyl)-4-(6-(4-(diethylamino)phenyl)hexatrienyl) pyridinium dibromide (FM4-64), with the fluorescence of RH-414 homogeneously distributed in the plasma membrane [90], while FM4-64’s fluorescence is concentrated in the actively recycling membrane at synaptic connections [91]. At cerebellar granule neurons (CGNs), R_0_ values for both pairs of donor–acceptor molecules were in the range of 3.7–4.2 nm, with a FRET efficiency of 30–35% at 9 days of cell culture (mature CGN). Fluorescence microscopy images of CGN stained with FM4-64 obtained at the donor wavelength excitation showed that FM4-64 is distributed in clusters or discrete membrane domains 0.5–1 μm in diameter, largely present at inter-neuronal contact sites of the neuronal soma [92].

The membrane-bound isoform of Cb5R is a major protein component of the plasma membrane redox chain in rat liver cells [93], one of the five more abundant proteins of the caveolin–protein complex isolated from the vascular endothelial membrane [94], and a flavoprotein. Thus, working with CGN, FRET studies were conducted between CTB-Alexa488 and anti-caveolin-2/IgG-Alexa488 (as lipid raft markers) and anti-Cb5R/IgG-Cy3. The observed FRET between CTB-Alexa488 and anti-Cb5R/Cy3 indicates that Cb5R must be present in most “lipid raft” domains of the plasma [95]. More recently, FRET imaging with cerebellar cortex slices using Alexa488-cholera toxin B as the donor and the complex anti-Cb5R isoform 3/IgG-Cy3 as the acceptor confirms that a large part of Cb5R isoform 3 is located vicinal to lipid raft nanodomains [96]. Further FRET studies using CGNs in culture, with a methodological improvement based on the implementation of secondary fluorescent antibodies (Alexa488-IgG and Cy3-IgG) directed against primary antibodies for Cb5R and L-type calcium channels (L-VOCC), led to the suggestion that L-VOCC was anchored to raft domains through binding to caveolin complexes. This could occur at a distance between 10 and 100 nm from cytochrome b5 reductase [97]. Similar FRET experiments showed an enhanced clustering of cytochrome b5 reductase within caveolin-rich lipid raft microdomains in the early phase of apoptosis [98]. A contemporary study using a great variety of donor–acceptor pairs indicated that (a) L-VOCC and N-methyl D-aspartate receptors (NMDARs) are vicinal proteins in the plasma membrane, separated by less than 80 nm; (b) NMDARs also colocalize with neuronal nitric-oxide synthase (nNOS); (c) nNOS is closer to caveolin-2 than caveolin-1; and (d) CTB binding sites were located extracellularly and nNOS sites intracellularly. Together, these findings suggested the clustering of NMDARs, L-VOCC, and nNOS in caveolin-rich microdomains with dimensions <100 nm. Gutiérrez-Merino and colleagues named these domains “calcium-microchip-like structures” and speculated that they have a high degree of control over the neuronal excitability by calcium [99]. The donor–acceptor pairs used were anti-L-VOCC/IgG-Alexa488 and anti-NMDAR/IgG-Cy3; anti-NMDAR/IgG-Alexa488 and L-VOCC ligand ST-BODIPY dihydropyridine, a much smaller molecule; anti-nNOS/IgG-Alexa488 and anti-NMDAR/IgG-Cy3; and anti-nNOS/IgG-Alexa488 and CTB-Alexa555 as donors and anti-caveolin-1/IgG-Cy3 and anti-caveolin-2/IgGCy3 as acceptors [99].

Additionally, co-localization of the calcium extrusion systems (PMCA, plasma membrane calcium pump, and NCX, sodium–calcium exchanger) with the major calcium entry systems (L-VOCC and NMDAr) and the ROS/RNS enzymes (Cb5R and nNOS) was observed within lipid raft-associated sub-microdomains smaller than 200 nm [100]. From these studies, lipid rafts emerged as interesting markers of plasma membrane nanodomains, where cross-talk between redox and calcium signaling occurs.

Recently, and as a continuation of the same approach, Gutiérrez-Merino and colleagues initiated a series of studies on Alzheimer’s disease, exploring the relationship between Aβ peptides, membrane lipids, and dysregulation of calcium homeostasis. FRET imaging was performed in mature CGN between the fluorescent derivative Aβ (1–42)-HiLyteTM Fluor555 and anti-CaM (calmodulin) conjugated with IgG-Alexa 488 (anti-CaM*A488) or anti-LTCC subunit 1C conjugated with IgG-Alexa 488 (anti-LTCCs*A488), and between anti-CaM*A488 and anti-HRas*Cy3. Several protein markers of lipid rafts were used as acceptors: anti-Cav-1 conjugated with IgG-Alexa 488 (anti-Cav1*A488), anti-HRas conjugated with IgG-Alexa 488 (anti-HRas*A488), and anti-PrPc conjugated with IgG-Alexa488 (anti-PrPc*A488). An extensive complexation of Aβ with CaM and LTCC with colocalization of CaM and HRas together with an Aβ association with lipid raft sub-microdomains was observed [101]. The dysregulation of calcium homeostasis observed in Alzheimer’s disease was attributed to the inhibition of LTCC by CaM–Aβ complexes. In a subsequent study, using the same fluorescence-labeled Aβ and Alexa488-labeled secondary antibodies for primary anti-PDI and anti-CaM or stromal interaction molecule 1 (STIM1) labeled with green fluorescent protein (STIM1-GFP), Gutiérrez-Merino and colleagues demonstrated that prior to the internal dysregulation of calcium homeostasis, a perturbation of store-operated calcium entry occurs through the internalization of Aβ and its inhibition of STIM1, along with partial activation of the ER Ca^2+^-leak channels [102]. For these measurements, variations in donor fluorescence were analyzed, and the R_0_ and the spectral overlap integral (J) values were obtained for the Aβ*555–SPM1-GFP donor–acceptor pair [102]. Recently, based on a similar formalism, Gutiérrez-Merino and colleagues showed that a short peptide of only six histidines binds with high affinity to Aβ(1–42) and also to Aβ(25–35), which still remains toxic, blocking the interaction of Aβ with CaM and calbindin-D28k [103]. This latest result opened an interesting line of research on the modulation of Aβ peptide toxicity.

## 5. Concluding Remarks and Future Prospects

In his “Zwischenmolekulare Energiewanderung und Fluoreszens (Intermolecular energy migration and fluorescence)” [28] and “Experimentelle und theoretische Untersuchung des zwischenmolekularen Übergangs von Elektronenanregungsenergie” [104] papers, Theodor Förster set the theoretical basis of what we currently know as the Förster resonance energy transfer (FRET) phenomenon, a non-radiative process through which a donor fluorophore in the excited state transfers energy to nearby acceptor molecules within nanometric distances. Lubert Stryer and Richard Haughland were among the first to qualify FRET as a “spectroscopic ruler” [105]. Gregorio Weber, another forefather of fluorescence spectroscopy in biological applications, considered FRET as the ultimate yardstick in inter-molecular and intra-molecular measurements in solution. Carlos Gutiérrez-Merino made ample use of FRET in his many studies on biological systems and contributed to setting the theoretical basis for the application of FRET in biological membrane research. During our decade-long collaboration with Carlos Gutiérrez-Merino, we conceived the use of FRET in combination with the so-called general polarization of Laurdan, a solvatochromic fluorescent probe designed and synthesized by Gregorio Weber [106]. This combination resulted in FRET-GP, the first application of Laurdan as a sensor of a membrane-embedded protein’s lipid microenvironment by excitation of the protein’s intrinsic fluorophores [52].

The emergence of autofluorescent proteins and protein chimeras with spectroscopic characteristics suitable for FRET applications has opened the way to implement Förster resonance energy transfer in the light microscope. The study of four-dimensional (x,y,z,t) molecular-scale phenomena in live cells [107] is thus a reality that now projects beyond the optical diffraction barrier and into the realm of super-resolution light microscopy [108,109,110,111,112,113,114]. Furthermore, the ability to genetically manipulate the chemistry of donor and acceptor fluorescent proteins has expanded the spectral coverage of fluorescence microscopy in live-cell interrogation [110]. These advances are particularly relevant to the field of membrane biology. Hybrid FRET models that incorporate fluorescence spectroscopy concepts, optical microscopies, and artificial intelligence approaches (computational tools such as deep learning and neuronal networks) are now reaching atomistic structural levels and temporal resolutions relevant to cell phenomena. Additionally, a new dimension of FRET has been opened with hybrid/integrative modeling, where bio-macromolecular systems are studied simultaneously through a combination of experimental techniques (such as small-angle neutron and small-angle X-ray scatterings, along with NMR, EPR, and FRET spectroscopies), as reviewed in [115]. Single-molecule fluorescence spectroscopy is an emerging field in which FRET is a necessary partner, as demonstrated in a recent study characterizing the coexistence of stable oligomers of amyloid-β 42 within a heterogeneous and dynamic sample [116].

## Data Availability

Not applicable.
